# Wild Bilberry (*Vaccinium myrtillus* L., Ericaceae) from Montenegro as a Source of Antioxidants for Use in the Production of Nutraceuticals

**DOI:** 10.3390/molecules23081864

**Published:** 2018-07-26

**Authors:** Snezana Brasanac-Vukanovic, Jelena Mutic, Dalibor M. Stankovic, Ivana Arsic, Nada Blagojevic, Vesna Vukasinovic-Pesic, Vanja M. Tadic

**Affiliations:** 1 Faculty of Metallurgy and Technology, University of Montenegro, Dzordza Vasingtona bb, 20000 Podgorica, Montenegro; sneza_b@yahoo.com (S.B.-V.); nadab@ac.me (N.B.); vesnav@ac.me (V.V.-P.); 2 Faculty of Chemistry, University of Belgrade, Studentski trg 12-16, 11001 Belgrade, Serbia; jmutic@chem.bg.ac.rs (J.M.); dalibors@chem.bg.ac.rs (D.M.S.); 3 The Vinča Institute of Nuclear Sciences, University of Belgrade, POB 522, 11001 Belgrade, Serbia; 4 Faculty of Medicine, Department of Pharmacy, University of Nis, 18000 Nis, Serbia; ivanafarmacija@gmail.com; 5 Institute for Medicinal Plant Research “Dr. Josif Pancic”, Tadeusa Koscuska 1, 11000 Belgrade, Serbia

**Keywords:** *Vaccinium myrtillus*, phenolic compounds, metals, antioxidant activity, cyclic voltammetry, principal component analysis

## Abstract

The aim of this study was to establish correlation of chemical composition and antioxidant activity of bilberry plants from Montenegro. Total phenolic, tannin, flavonoid, procyanidin and anthocyanin contents were determined in fruits and leaves extracts using spectrophotometric methods, while the measurements of metal content was carried out in an Inductively Coupled Atomic Emission Spectrometer. Qualitative and quantitative analyses of major phenolics were achieved by HPLC. In the investigated extracts, the most abundant phenolic was chlorogenic acid, followed by protocatechuic acid, while resveratrol, isoquercetin, quecetin and hyperoside were also present in significant quantities. Antioxidant potential was evaluated using two in vitro assays—FRAP and DPPH—being in the accordance with the cyclic voltammetry tests, performed as well. The results revealed that all the investigated extracts were rich in phenolic and essential mineral constituents, with significant antioxidant activity, depending on the polyphenolic and mineral contents, which was confirmed by principal component analysis.

## 1. Introduction

Phenolic compounds are the most abundant secondary metabolites of plants, with more than 8000 phenolic structures ranging from simple molecules such as phenolic acids to highly polymerized substances such as tannins described. It is known that the antioxidant activity of phenolics is mainly due to their redox properties, which allow them to act as reducing agents, hydrogen donors, and singlet oxygen quenchers [1]. A wide variety of phenolic compounds may help protect cellular systems from oxidative damage [2]. The increased consumption of plant naturally rich in phenolics might be associated with prevention of cardiovascular diseases, diabetes, cancer, and obesity. Apart from beneficial human health effects, the phenolic compounds present in plant tissues take active part in plant protection against bacterial and fungal infections, UV radiation, physical damage, etc.

Phenolic compound consumption is associated with lower risk for chronic disease development. They can inhibit α-glucosidase and α-amylase activities, which are the key enzymes responsible for the digestion of dietary carbohydrates to glucose. Polyphenols take a part in modulating carbohydrate and lipid metabolism, they might attenuate hyperglycemia, dyslipidemia and insulin resistance, stimulate insulin secretion, improve β-cell function, adipose tissue metabolism and alleviate oxidative stress, stress-sensitive signaling pathways and inflammatory processes. These compounds can also prevent the development of long-term complications of diabetes, including cardiovascular disease, neuropathy, nephropathy and retinopathy [3].

*Vaccinium myrtillus* L. (bilberry) is a member of the Ericaceae family. Bilberry is one of the most trusted herbal medicines for treating diabetes [4]. In a survey of 685 Italian herbalists, bilberry ranked fourth in a list of herbal remedies recommended for improvement of glycemic control [5]. Traditionally, people have used bilberry leaves to control blood sugar, what is might be ascribed to various phenolic compounds, being flavonoids, phenolic acids, tannins and stilbenes the most importnt. On the other hand, the fruits do not lower blood sugar, but their constituents may help improve the strength and integrity of blood vessels and reduce their damage, which is associated with diabetes, among the others diseases. This effect is due to anthocyanins which represent nearly 90% of the total phenolics. In addition, phenolics compounds might be responsible for the high antioxidant potential reported for bilberry [6]. The antidiabetic potential of bilberry can be attributed to the mineral elements present in it, as well [7,8,9].

The literature data reveals that the concentration and the profile of phenolic compounds in plant extracts depends mostly on the polarity of solvents used in the extraction process, on the different plants’ biological habitat and growing conditions, as well as on the nature of the associations that phenolics might form with other plant components such as carbohydrates and proteins [10]. Although bilberry fruits have been described elsewhere in terms of their chemical profile and/or their antioxidant capacities, there is not much research focusing on the correlation between the investigated antioxidant properties and chemical profile of different types of bilberry leave extracts. Cyclic voltammetry is a useful analytical procedure for fast monitoring of antioxidant activity of different kind of samples. The electrochemical techniques developed for antioxidant capacity screening and assessment have proved rapid and sensitive, allowing analysis of various matrices, with no need for complicated sample pre-treatment and with relatively low cost. The methods exploit either the peak current intensity or the value of the charge, obtained from the area below the current wave labelled as antioxidant capacity [11].

In choosing the suitable solvent for extraction several factors should be taken into account. Namely, the non-covalent interactions, the speed of the molecule movements and acceleration of the solvent flow at higher temperature might increase the content of extracted phenolic compounds [12]. On the other hand, the temperature at the boiling point of the used solvent and the decrease of fluid density at higher temperature can reduce the extraction efficiency of phenolic compounds. Therefore, we used different extraction methods and solvents with different polarity to establish suitable conditions for obtaining extracts rich in polyphenolic composition that might be satisfactory in providing the good effects traditionally established.

In this context, the goal of the current study was to establish correlation of chemical composition and antioxidant activity of the bilberry fruits and leaves extracts obtained by infusion, maceration and Soxhlet extraction. Taking into account that water herbal infusions are most often used for preparation of traditional phytopreparations during times of illness, but according to the fact that industrial scale production usually uses ethanol as solvent we also used Soxhlet extraction as a model method of a complete exhaustive extraction of the plant material. In addition, bearing in mind that Soxhlet extraction requires high temperature, what might be disadvantageous for thermolabile substances, maceration was employed, as well, as extraction on ambient temperature. Hence, the aims of this study were to determine total phenolic (TP), total tannins (TT), total flavonoid (TF) content and the mineral composition in the bilberry leaves and fruits extracts. In order to identify and quantify major phenolic compounds present in the investigated samples a HPLC method was employed. To assess the relationship between antioxidant activity and phenolic compounds content, as well as the metals content of investigated extracts, two common antioxidant activity assays—DPPH and FRAP—were applied. In addition, cyclic voltammetry was performed, as well, as a reliable method for antioxidant properties estimation. To establish the correlation between the chemical composition and the antioxidant properties of the investigated extracts, principal component analysis was employed.

## 2. Results and Discussion

### 2.1. Yield, Content of Polyphenolic Compounds and Mineral Composition in the Extracts

#### 2.1.1. Yield of Extractions

The yield of extraction from plant material depends on the type of solvents with varying polarities, extraction time and temperature, sample-to-solvent ratio as well as on the chemical composition and physical characteristics of the sample components. For the preparation of the investigated extracts water and 70% ethanol were chosen, as they might be considered environmentally benign and relatively safe to human health. Although Pop et al. [13] showed that when 80% ethanol was used for extraction it provided the highest yield, we employed 70% ethanol in our investigation, bearing in a mind that extraction at the industrial scale is usually performed with this solvent. Taking into consideration that the traditional applications involve water infusions, the second solvent used in our experiment was water [14].

**Figure 1:**
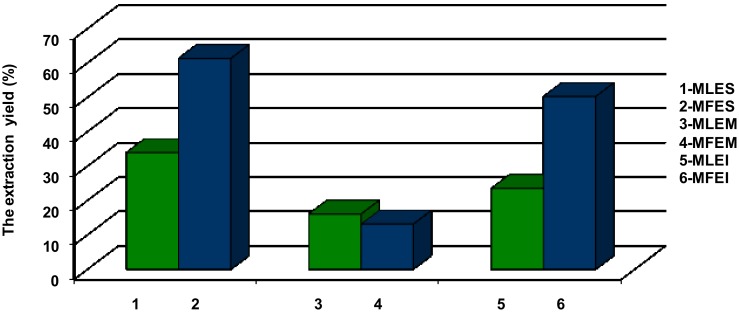
The extraction yields (%): MLES—*Myrtilli folium* extract obtained by Soxhlet; MFES—*Myrtilli fructus* extract obtained by Soxhlet; MLEM—*Myrtilli folium* extract obtained by maceration; MFEM—*Myrtilli fructus* extract obtained by maceration; MLEI—*Myrtilli folium* extract obtained by infusion; MFEI—*Myrtilli fructus* extract obtained by infusion.

Based on the results shown in Figure 1, the extraction yield was highest when 70% ethanol was employed in a Soxhlet extraction (MFES 61.35%, MLES 33.96%). Significantly lower yields were obtained with the same solvent, but applying maceration (MFEM 13.10%, MLEM 16.09%). The extraction yield obtained with water as a solvent, was higher than the yield achieved with maceration (with 70% ethanol), but lower in comparison to Soxhlet extraction and 70% ethanol (50.25% and 23.51% in MFEI and MLEI, respectively). The advantage of the Soxhlet extraction compared to the cold maceration using the same solvent indicated that increasing the extraction temperature might increase the solubility of extractives, leading to an increase in yield as well. The better yields of dry matter resulting from Soxhlet extraction with 70% ethanol compared to extraction of water were probably a consequence of the circulation flow of the solvent up to the complete exhaustion of plant material. Better extraction yield when using water extraction compared to maceration with ethanol, could be the result of lower selectivity of water as a solvent in which, besides the desired polyphenolic components, other components such as proteins and carbohydrates were dissolved too.

#### 2.1.2. Total Phenolic Content

**Figure 2:**
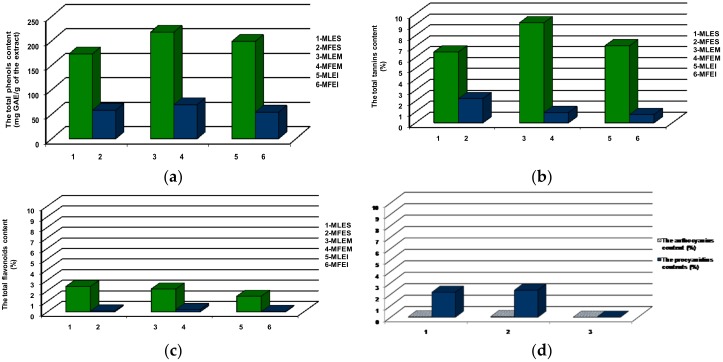
Content of (**a**) total phenolic compounds (TP); (**b**) total tannins (TT); (**c**) total flavonoids (TF); (**d**) total procyanidins and total anthocyanins in the investigated extracts.

In this study we investigated the presence of phenolic compounds, known from the up to date literature data to possess antidiabetic potential [15,16]. In our study (Figure 2a), TP content found in leaves extracts was in the range from 173.19 to 217.59 mg GAE/g (in MLES and MLEM, respectively), and in fruits extracts it ranged from 53.95 to 69.32 mg GAE/g (MFEI and MFEM, respectively), which were in line with the TP content found by Vučić et al. [17]. In our samples, TT (Figure 2b) content in the leaves (from 6.49% in MLES to 9.17% in MLEM) and fruits (from 0.76 in MFEI to 2.21% in MFES) extracts were in good agreement with the values obtained by Roslon et al. [18]. The TF (Figure 2c) content in the fruits extracts varied from 0.02% in MFEI to 0.21% in MFEM, while in the leaves extracts, TF represented 1.46%, 2.15% and 2.38% of MLEI, MLEM and MLES, respectively. The literature data revealed that the concentration of flavonoids in the bilberry leaves and fruits extracts was higher in ethanol than water extracts [17]. In addition, the same authors found that the ethanol and water leaves extracts were significantly richer in TP and TF compounds in comparison to the fruits extracts. The data presented in this study were similar to the cited literature, having in a mind that some other literature data pointed out that average flavonoids content in bilberry leaves extracts were significantly lower [18]. In the case of procyanidins and anthocyanins (Figure 2d) in the fruits extracts, the highest content was found in MFEM (2.36% and 0.06%, respectively) while the lowest was seen in MFEI. The determined low anthocyanins content in the investigated fruits extracts was in accordance to the published data stating that the content of anthocyanins declined after drying [18]. The significant difference in TP, TT, TF content between all extracts was observed (*p* < 0.01). Also, in procyanidins and anthocyanins content significant difference in fruits extracts was found (*p* < 0.01).

#### 2.1.3. HPLC Analysis

**Figure 3:**
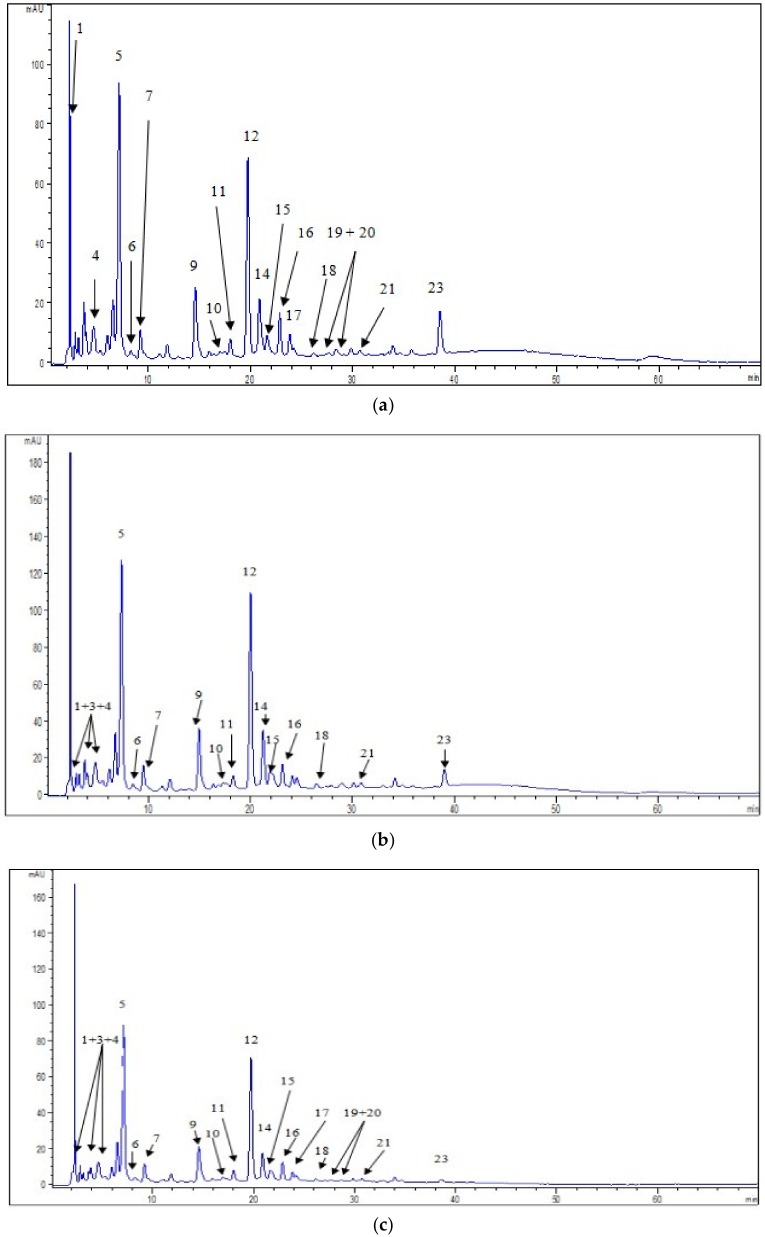

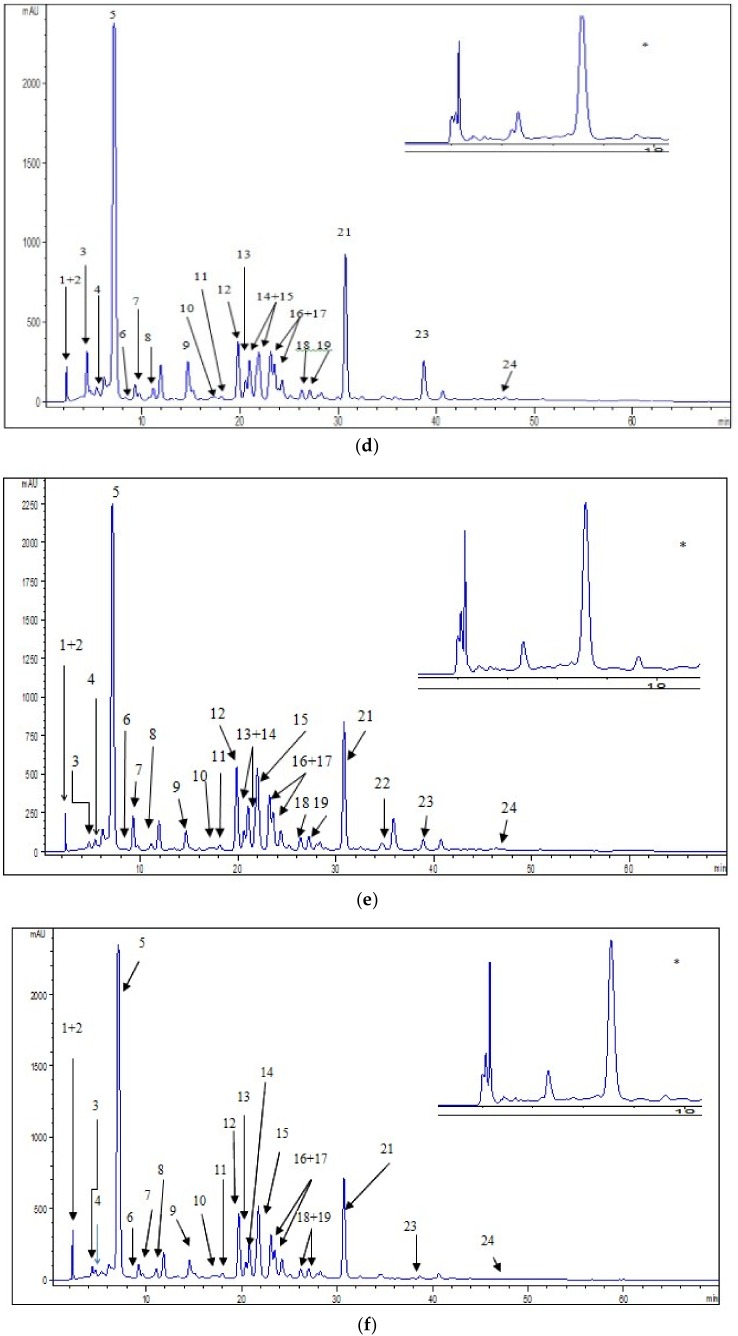
HPLC chromatograms of investigated extracts and phenolic compound identified at 325 nm in: (**a**) MFES; (**b**) MFEM; (**c**) MFEI; (**d**) MLES; (**e**) MLEM; (**f**) MLEI. (* Separation of gallic acid and pyrogallol was also presented at 260 nm in MLES, MLEM and MLEI).

In our investigation we confirmed the presence of eighteen individual phenolic compounds and six non-identified derivatives of quercetin, chlorogenic acid and stilbenoids (Figure 3, Table S1 in the Supplementary Material). The identification of the mentioned phenolic compounds was important taking into account that some phenolics could reduce the risk of type 2 diabetes and the associated increased risk of microvascular and macrovascular effects [15,16]. The presence of individual phenolic compounds in bilberry has been reported in several studies [2,5,6,14,19,20]. Different contents of phenolic compounds might be explained by the influence of different abiotic environmental factors (temperature, water deficiency, irrigation, and nutrient stress) or the different extraction procedures employed. 

The presence of phenolic acids in the investigated extracts (Figure 3, Table S1 in the Supplementary Material) was significant. This finding might be important according to the study performed by Bahadoran et al. [21], which revealed that phenolic acids stimulated glucose uptake with comparable performance to metformin and thiazolodinedione, main common oral hypoglycemic drugs. The predominant phenolic acid in the leaves extracts was chlorogenic acid (from 45.51 in MLEM to 59.70 mg/g in MLEI) also identified in fruits extracts, with contents ranging from 1.82 to 2.48 mg/g, in MFEI and MFEM, respectively. Protocatechuic acid was detected in all leaves and fruits extracts (from 1.40 in MLEM to 1.74 mg/g in MLEI and from 1.10 in MFEI to 1.42 mg/g MFEM). Determined polyphenolc acids, present in all investigated extracts, but in lower amounts, were gallic, caffeic, *p*-coumaric, sinapic and ferulic acid. Neochlorogenic acid was not detected in MFES. The content of chlorogenic, *p*-coumaric, caffeic, gallic and ferulic acids in our fruits extracts were higher compared to previously reported data for fruits originated from Slovenia [6]. We found a significant difference (*p* < 0.01) between all the extracts in all identified phenolic acids content, except in the case sinapic acid between MFES and MFEI. The antidiabetic potential of phenolic acids might be ascribed to their modulatory effects on glucose transporter (GLUT) by enhancing GLUT2 expression in pancreatic β-cell and increasing the expression and promoting the translocation of GLU4. Based on the literature data, phenolic acids might stimulate the influx of Na^+^ and Ca^2+^ ions by inhibiting the Na^+^-Ca^2+^ exchanger, thereby stimulating the influx of Ca^2+^ into the inner membrane, leading to membrane depolarization and activation of the synthesis of adenosine triphosphate through oxidative phosphorylation. This mechanism comprised the stimulation of insulin secretion [15].

In the case of flavonoids (Figure 3, Table S1 in the Supplementary Material), in the leaves extracts we detected isoquercetin (from 9.92 in MLES to 16.20 mg/g in MLEI), quercetin (from 1.16 in MLEI to 7.27 mg/g in MLES), rutin (from 4.73 in MLEM to 5.31 mg/g in MLEI), hyperoside (from 2.38 in MLES to 2.55 mg/g in MLEI), kaempferol-3-*O*-glucoside (from 1.38 in MLES to 1.60 mg/g in MLEI) and kaempferol (from 0.03 in MLEI to 0.26 mg/g in MLES). The significant difference in these flavonoids content was found between leaves extracts (*p* < 0.01). Quercetin (from 0.07 in MFEI to 0.46 mg/g MFES), hyperoside (from 0.17 in MFEI to 0.34 mg/g in MFEM), kaempferol-3-*O*-glukoside (from 0.05 in MFEI to 0.15 mg/g in MLEM) and isoquercetin (to 0.31 mg/g) were found in fruits extracts. All mentioned flavonoids content showed significant difference between fruits extracts (*p* < 0.01), except in isoquercetin content between samples MFEM and MFEI. The determined content of quercetin in the leaves and fruits extracts was higher than results obtained by Hokkannen et al. [22] and Može et al. [6]. Kaempferol was detected in our leaves extracts which was in accordance with the date obtained by Bljajic et al. [19], while in the work of Hokkanen et al. [22] this compound was not found. Rutin was not present in our fruit samples, while this flavonoid was detected in Slovenian fruits [6]. Piparo et al. [23] showed that the potency of inhibition of human α-amylase, responsible for the digestion of dietary carbohydrates to glucose, was correlated with the number of hydroxyl groups on the B ring of the flavonoid skeleton. The results from the in vitro studies showed that quercetin, isoquercetin and hyperoside stimulate GLUT4 translocation and expression in skeletal muscle, by mechanisms associated with the activation of 5′-adenosine monophosphate-activated protein (AMPK) [16]. Effect of polyphenols in activation of AMPK, as a main target for anti-diabetic drugs, has been reported to be 50–200 times more than metformin [21]. Rutin and kaempferol also stimulate glucose uptake. Taking into account the presence of the mentioned flavona and flavonols in our samples, for which it was shown to possess capability to interact with the enzymes responsible for digestion of carbohydrates, the assumption was that the traditionally employed bilberry in prevention and cure of diabetes mellitus might have its justification.

In our study, epicatechin was only found in the leaves extracts (from 4.38 in MLEI to 5.75 mg/g in MLES) which was in accordance with the literature data [22]. Previously investigation revealed it presence also in the bilberry fruit [6]. Pyrogallol was detected only in the leaves extracts (from 2.45 in MLEM to 3.46 mg/g in MLES). The significant difference between extracts of leaves in epicatechin and pyrogallol content (*p* < 0.01) noticed. Our investigation revealed the presence of procyanidin in the both, extracts of leaves and fruits, as well (Figure 3, Table S1 in the Supplementary Material). There was a significant difference in procyanidin content in all investigated extracts (*p* < 0.01). Epicatechin, pyrogallol and procyanidins were known to have ability to protect β-cells from hyperglycemia and oxidative damage. Oral administration of phenolics had favourable effects on serum glucose and viability of β-cell through attenuation of oxidative stress, enhancing the natural antioxidant system, and inhibition of lipid peroxidation [21]. Although checked for presence in this study, we did not detect catechin, as it was published that bilberry also contained this compound [6,22].

Stilbenes were detected in all extracts of bilberries (Figure 3, Table S1 in the Supplementary Material). The content of resveratrol in leaves extracts was from 4.60 in MLEM to 5.15 mg/g in MLES, while in fruits it was found to vary 0.01 in MFEI to 0.07 in MFEM. The significant difference between all the extracts in resveratrol content noticed (*p* < 0.01). There are no reports of the presence resveratrol in bilberry leaves. However, previous data for resveratrol content in bilberry fruits was lower than ours [6]. Some experimental and in vitro studies demonstrated that resveratrol has the potential to improve glucose tolerance, attenuate β-cell loss, and reduce oxidative stress in pancreatic islet [21].

#### 2.1.4. Metal Content

According to our results, the water leaves and fruits extracts (MFEI and MLEI) contained higher amount of Mn, Zn, K, Mg, Ca, Cr and Cd than ethanol extracts. On the other hand, it was determined the higher content of Cu and Pb in ethanol in comparison to the water extracts. The water fruits extracts contained significant quantity of Fe and As, while in leaves, those metals were present in highest quantity in MLEM (Table S2a in the Supplementary Material). Based on the literature data, the different quantity of metals determined in the investigated extracts might be dependent on the extraction medium. Besides, the duration and temperature of the extraction process highly influenced the metals profile and their quantity. The content of metals (Table S2 in the Supplementary Material) in leaves extracts were similar to the results obtained for the leaves extracts originated from North and Middle European countries investigated by Reimann et al. [9] and by Kozanecka et al. [8]. In addition, the concentration of metals in our fruits extracts was in good agreement with the results presented by Demczuk and Garbiec [7].

The significant presence of Cr, Mg, Zn, Mn, Fe, Cu, K and Ca in the bilberry investigated samples might be of great importance in connection with traditional applications of bilberry leaves and fruits as drugs with antidiabetic potential. Direct associations of macro and trace elements with diabetes mellitus have been observed in many research studies [24,25]. Insulin action on reducing blood glucose was reported to be affected by some essential elements as Cr, Mg, V, Zn, Mn, Mo, Se, Fe, Cu, K and Ca. The proposed mechanism of metals enhancing insulin action includes activation of insulin receptor sites, serving as cofactors or components for enzyme systems involved in glucose metabolism, increasing insulin sensitivity, and acting as antioxidants preventing tissue peroxidation. It has also been reported that the metabolism of several trace and macro elements alter diabetes mellitus and these elements might have specific roles in the pathogenesis and progress of this disease [24]. In a case of essential elements deficiency and increased exposure to toxic metals such as Pb, Cd and As, body tends to use toxic instead of essential elements. These toxic metals are elevated in biological samples of diabetic patients, which adversely affect health status of an individual by disrupting organ physiology and functions [25]. 

The highest concentration of K, Ca and Mg were detected in MLEI, while the lowest were in MFEM. The concentration of K was from 6.01 ± 0.10 to 17.22 ± 0.02 mg/g, Ca from 0.383 ± 0.005 to 5.94 ± 0.02 mg/g, and Mg from 0.397 ± 0.007 to 3.83 ± 0.02 mg/g. There was significant difference between all the extracts in Mg, Ca and K content (*p* < 0.01). These macro elements improved insulin sensitivity, responsiveness and secretion of insulin. Any alterations in Ca flux can have adverse effects on β-cell secretory function while K depletion can result in reduced glucose tolerance [24]. Mg is a cofactor in the glucose transporting mechanisms of the cell membrane and various enzymes in carbohydrate oxidation. It is also involved at multiple levels in insulin secretion, binding and enhancing the ability of insulin to activate tyrosine kinase [25]. Therefore, our results indicated that the significant quantity of the mentioned macroelements in the investigated extracts might have beneficial effects in metabolic process impairments.

In our study, the concentrations of Mn varied from 42.85 ± 0.10 in MFES to 1210 ± 2 µg/g in MLEM, Fe from 5.90 ± 0.05 in MFEM to 37.6 ± 0.2 µg/g in MFEI, Cu from 1.513 ± 0.010 in MFEI to 33.31 ± 0.09 µg/g in MLES, Zn from 6.281 ± 0.007 in MFEM to 31.48 ± 0.04 µg/g in MLEI, and Cr from 0.123 ± 0.002 in MLEM to 1.11 ± 0.03 µg/g in MLEI. There is a significant difference between all the extracts in the content of these essential metals (*p* < 0.01). Mn is a cofactor of pyruvate carboxylase, which plays a role in the conversion of various non-carbohydrate compounds into glucose via gluconeogenesis for their subsequent use [25]. Cr regulates insulin and blood glucose levels by stimulating insulin signalling pathways and metabolism by up-regulating GLUT4 translocation in muscle cells while Cu activates cytochrome oxidase which is involved in the electron transport chain of the mitochondria [25]. It was established that Zn transporter is a key protein involved in the synthesis and secretion of insulin from the pancreatic β-cells [25]. The health benefits derived from these mineral elements is a clear indication that their deficiencies appear to be an additional risk factor in the development and progress of disease and they contribute to the pathogenesis of diabetes mellitus and its complications. Their repletion may be an effective therapeutic intervention in prevention of the progression of the diabetes and its complications, along with a glycemic control and control of other risk factors. This study, therefore, might help in getting the better insight into antidiabetic plants effect as glucose lowering agents because of the shown appreciable quantities of the determined mineral elements associated to the investigated extracts.

The toxic metals Pb, Cd and As determined in our samples might be additional risk factor in the development and progress of diabetes mellitus and its complications. The permissible limit for Cd and Pb in medicinal plants set by World Health Organization (WHO) were 0.3 µg/g and 10 µg/g, respectively, having in a mind that for As a limit has not yet been established by the WHO [26]. The calculated values showed that all investigation extracts accumulate these metals below the limits (Table S2a in the Supplementary Material). Based on our findings, the extracts of bilberry possessed no toxicological risk. There was significant difference between all the extracts in content of Pb (*p* < 0.01). On the other hand, we did not find significant difference in As content between MFES and MFEM, and in Cd content between all fruits extracts.

Taking into account that the average consumption of leaves/fruits extracts obtained by infusion/Soxhlet extraction/maceration, and the obtained average yield (Figure 1), we took 1 g of bilberry fruits and leaves extracts to calculate daily mineral intakes (DMI) according to the European Economic Community norms [27]. The presented results revealed that the investigated extracts, could be prospective source, not only of antioxidant phenolics (Figure 2), but of Mn (Table S2b, Supplementary Material), but a nutraceuticals/functional food as well. Besides, based on our findings (Table S2b, Supplementary Material), the extracts of bilberry possessed no toxicological risk.

### 2.2. Antioxidant Activity

Reactive oxygen species (ROS) and reactive nitrogen species (RNS) are highly reactive oxidized molecules, which are generated constantly by normal cellular process like the activity of the mitochondrial respiratory chain and inflammation. ROS include free radicals such as superoxide (O^2−^) and hydroxyl (OH), and non-free radicals such as hydrogen peroxide (H_2_O_2_). Reactive nitrogen species include free radicals such as nitric oxide (NO) and nitrogen dioxide (NO_2_^−^), and non-free radicals such as peroxynitrite (OONO^−^). Oxidative stress can be defined as any disturbance in the balance of antioxidant and pro-oxidants and it occurs due to an increased generation and/or reduced elimination of reactive species (RS) by the antioxidant defense system. To prevent oxidative damage, it is important that excess RS be eliminated from the cells. The ability of cells or tissues to withstand oxidative stress is largely dependent on the efficiency of the overall antioxidant defense system to scavenge excess RS, without compromising the physiological roles of ROS [28]. The antioxidant enzymes include superoxide dismutase (SOD), glutathione peroxidase (GPx) and catalase (CAT), all of them playing a vital role in quenching these oxidants and preventing cellular injury. The level of these antioxidant enzymes is associated with the development of complications in diabetes. This is particularly relevant and dangerous for the beta islets which have the lowest levels of intrinsic antioxidant defenses [29]. Plant polyphenols as dietary antioxidants can normalize the concentration of enzymes participating in carbohydrate metabolism and thus may prevent the oxidative changes [19].

**Figure 4:**
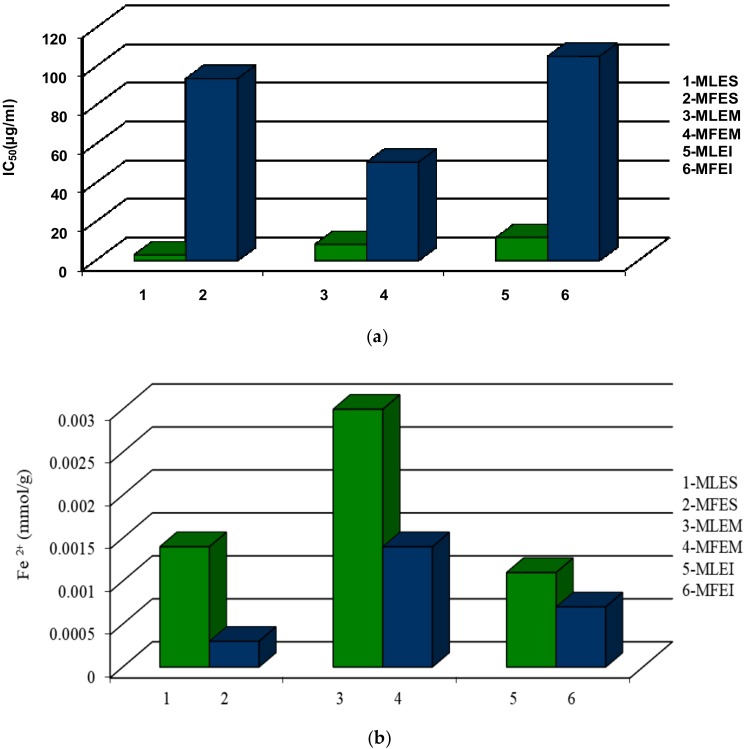
Antioxidant activity in the leaves and fruits extracts measured by (**a**) DPPH assay; and (**b**) FRAP test.

To evaluate the antioxidant properties, two different assays were employed to avoid any possible incorrectness in the interpretation of the obtained results regarding the antioxidant properties of the investigated extracts, having in mind the differences in their working principles. The antioxidant potential of all extracts was evaluated using the DPPH and FRAP tests. The DPPH assay is based on the hydrogen donating capacity to scavenge DPPH radicals, while the FRAP assay is an electron transfer-based test measuring the substance ability to reduce Fe^3+^ to Fe^2+^ (results were expressed as mmol Fe^2+^/g of extracts used). The ethanol extracts compared to water extracts had a higher antioxidant activity measured by DPPH assay (Figure 4a). IC_50_ values of the ethanol leaves extracts ranged from 2.92 to 11.94 µg/mL for MLES and MLEI, respectively. The fruits extracts had IC_50_ values in the range of 50.82 to 105.46 µg/mL, for MFEM and MFEI, respectively. The activities of the leaves extracts were similar to positive control, BHT (IC_50_ = 6 µg/mL), meaning that the investigated extracts possessed high antioxidant capacity. The investigated fruits extracts had lower antioxidant potential measured by the employed tests in comparison to leaves extracts (Figure 4a). However, the evaluated antioxidant activity of all investigated extracts was higher in comparison to early published data [2]. In the case of FRAP assay, (Figure 4b), the FRAP values was lowest for MLEI (0.0011 mmol/g) and the highest for the MLEM (0.0030 mmol/g), while in fruits extracts the FRAP values ranged from 0.0003 mmol/g for MFES to 0.0014 mmol/g for MFEM, what was lower than cited in the literature [5]. 

**Figure 5:**
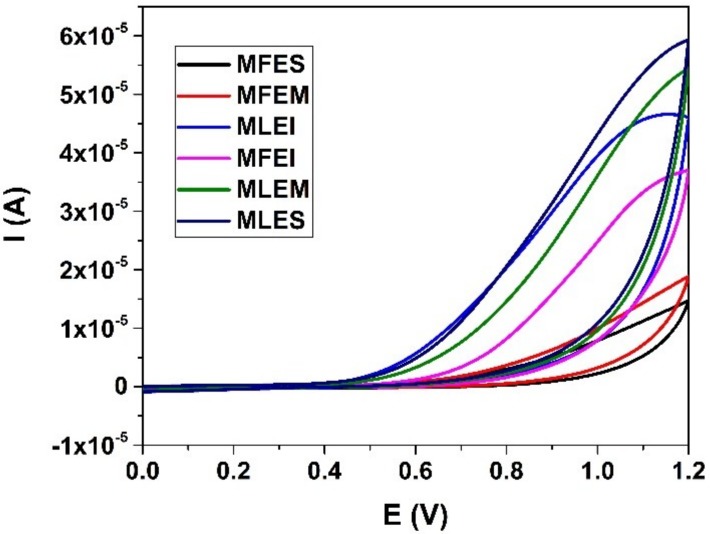
Cyclic voltammograms of samples. Working electrode BDD electrode, PBS at pH 7 as supporting electrolyte.

We found significant difference between all the extracts in DPPH values (*p* < 0.01), while in the case of FRAP values significant difference was between MLES-MLEM, MLEM-MLEI, MFES-MFEM. For estimation of the antioxidant activity of ethanol and water extracts of the samples cyclic voltammetry was employed as well. Representative voltammograms are shown in Figure 5. All samples provided one well defined oxidation peak in potential window between 0 and 1.2 V at an interestingly high potential. Absences of any corresponding cathodic peaks were observed. The performed measurements indicated that samples MFEM and MFES possessed lowest antioxidant capacity and can be grouped as a similar group of samples. Similarity was observed with chromatographic measurements (Table S1 in the Supplementary Material) where the absence of dominant phenolic compound was noted. On the other hand, samples labelled as MLEI, MFEI, MLEM and MLES showed similar behaviour using CV and higher antioxidant activity. The presence of one well defined peak for the MLEI, MLEM and MFES samples could be attributed to the higher presence of phenolic acids [30]. This was in accordance with results obtained with chromatographic measurements (Table S1 in the Supplementary Material). As antioxidant activity with electrochemical measurements was expressed as area under oxidation curve where usually one of two components provided visible and defined oxidation peaks and to the area under curve all components had a contribution [11]. Based on the results presented in Figure 5, the antioxidant capacity of the samples might be approximately estimated. Obviously highest antioxidant capacities were found in the samples labelled as MLEI, MLEM and MLES which was in good accordance with the measurements of total phenolic content and DPPH (Figure 2a and Figure 4a). On the contrary, lowest results from electrochemical measurements were obtained for samples MFES and MFEM. Similarly, same results were obtained with total phenolic content test for the same samples. As could be seen electrochemical measurements for sample MFEI revealed a medium antioxidant capacity value. This was not in accordance with other test measurements what might be explained by the nature of the sample and selection of used tests.

### 2.3. Correlation between Content of Polyphenolic Compounds and Mineral Composition and Antioxidant Activity

Principal component analysis (PCA) was performed to analyse antioxidant activity of the leaves and fruits extracts on the basis of their polyphenolic and mineral composition and to summarize obtained results (Figure S2a,b in the Supplementary Material).

We found a strong correlation between the DPPH and FRAP values (Figure S2b and Table S3 in Supplementary Material) for the fruits extracts (R^2^ = 0.7108) while in leaves extracts (Figure S2a and Table S3 in the Supplementary Material) the same correlation was very weak (R^2^ = 0.0006).

The explanation might be connected with the chemical profile of the investigated extracts, where the different compounds might contribute to antioxidant potential through different mechanisms [31].

The correlation between TF content (Figure S2a and Table S3 in the Supplementary Material) in leaves and antioxidant activity measured by the DPPH test (R^2^ = 0.8466) was strong, while the correlation with FRAP assays was poor, being only R^2^ = 0.1719. In the case of fruit extracts, the correlations between TF content and performed DPPH and FRAP assays were strong (R^2^ = 0.996 and R^2^ = 0.6517 respectively). TT contents in the leaves extracts exhibited a strong correlation with FRAP (R^2^ = 0.882), while it was weak in the case of DPPH values (R^2^ = 0.104). The poor correlations between TT content in fruits and both DPPH and FRAP values were established (R^2^ = 0.4898, R^2^ = 0.0434, respectively). Stronger correlation was determined for anthocyanins and procyanidins contents (fruits extracts, Figure S2b and Table S3 in the Supplementary Material) with DPPH (R^2^ = 0.8381; R^2^ = 0.5059, respectively) than FRAP values (R^2^ = 0.3085; R^2^ = 0.0491, respectively). TP content (Figure S2b and Table S3 in the Supplementary Material) in fruits extracts showed the strong correlation with DPPH assays (R^2^ = 0.9972), whereas with FRAP assays correlation was weaker (R^2^ = 0.6617). We found (Figure S2a and Table S3 in the Supplementary Material) a moderate correlation between TP and DPPH (R^2^ = 0.4529) and FRAP (R^2^ = 0.5249) values in the leaves extracts. In the samples for which a strong correlation was observed, it might be concluded that phenolics were largely responsible for the antioxidant activities observed within the samples. According to some previous studies, it was considered that hydroxyl groups present in the structure of the phenolic compounds might contribute to the oberved antioxidant activity [32,33]. However, the samples in which strong correlations between the TP content and antioxidant activity were not observed, there were probably significant amounts of antioxidants other than the measured phenolics present in the system. In addition, the antioxidant capacity also might be dependent on the structure and interaction between extracted phenolic compounds [34]. Namely, it is known that minerals, especially iron, can complex with phenolic compounds, influencing their antioxidant activity [35].

The antioxidant activity of phenolic acids depends on the number and positions of the hydroxyl groups in relation to the carboxyl functional group. It increases with increasing degree of hydroxylation, as it was the case of the trihydroxylated gallic acid [32], which showed a high level of antioxidant property (Table S4 in the Supplementary Material). The identified phenolic acids in the extracts of leaves and fruits mostly had a good correlation with DPPH or FRAP antioxidant activity (R^2^ was from 0.5641 to 1). Interestingly, in spite of the expectation that caffeic acid has higher antioxidant activity because of 3,4-position of dihydroxylation on the phenolic ring and additional conjugation in the propenoic side chain, which by resonance might facilitate the electron delocalization between the aromatic ring and propenoic group [33], the obtained results indicated a weak correlation between caffeic acids content in the investigated samples of fruits extracts and antioxidant activity (R^2^ < 0.5). Another key point to consider here might be the possible synergistic or antagonistic effects that could occur within the system based on additional components, as well as interactions between the phenolic compounds and their physical environment of the plant matrix [36].

The identified flavonoids derivatives (Table S4 in the Supplementary Material) in the extracts of leaves and fruits showed a strong correlation with DPPH or FRAP (R^2^ was from 0.7347 to 0.9903). Bearing in mind that the combination of the catechol moiety with double bonds at C2-C3 and 3-OH, quercetin and kaempferol might provide an extremely active free-radical scavenger agents [32]. In our study, quercetin from the leaves extracts exhibited strong correlation (R^2^ = 0.9355) with antioxidant properties. We found that resveratrol in all investigated extracts (Table S4 in the Supplementary Material) had strong correlation with DPPH (R^2^ was 0.7266 to 0.998). Resveratrol demonstrates an unusually strong ability to capture free radicals. This property is related to the presence of three hydroxyl groups, as well as the presence of aromatic rings and a double bond in the molecule [37]. Experimental studies confirmed that removal of hydroxyl groups or their replacement with -OCH_3_ groups results in a loss of antioxidant properties of the compound. We found strong correlation between pyrogallol with FRAP assay (R^2^ was from 0.8095). Pyrogallol and gallic acid conjugated derivatives, due to their higher molecular weight and high degree of hydroxylation of aromatic rings, might exhibit high antioxidant potential [38], confirmed by the found correlation with the determined antioxidant potential.

The stronger correlation of determined individual metals (Table S5 in the Supplementary Material) in the fruits extracts was established for DPPH values in comparison to FRAP values. The same was in the case of Mn, Cu, Mg, Ca, K and Pb contents found in the leaves extracts, while As, Cd, Ni, Cr, Zn and Fe exhibited strong correlation with FRAP values. In the leaves extracts (Table S5 in the Supplementary Material), the detected metals (apart from Pb) had strong correlations with antioxidant activities determined by both the DPPH and FRAP tests (R^2^ was from 0.7016 to 0.9997), while the most metals from the fruits extracts had moderate or weak correlations (R^2^ was from 0.1599 to 0.672).

We found strong correlation (Figure S2a and Table S6 in the Supplementary Material) between the total content of essential metals in leaves extracts and the antioxidant activity measured by the DPPH test (R^2^ = 0.8563), while in the fruits extracts the correlation was moderate (R^2^ = 0.5219). On the other hand, a high correlation was observed between toxic metals determined in the leaves extracts and FRAP values (R^2^ = 0.8933), while correlation of toxic metals content in fruits extracts with FRAP and DPPH was similar (R^2^ = 0.9221).

## 3. Materials and Methods

### 3.1. Materials

#### 3.1.1. Chemicals

Analytical grade reagents acetate buffer, 2,4,6-tripyridyl-s-triazine (TPTZ), HCl, FeCl_3_, Folin-Ciocalteu reagent,2,6-di-*tert*-butyl-4-methylphenol (BHT), *n*-butanol (BuOH), acetone, ethyl acetate, sodium bicarbonate, 1,1′-diphenyl-2-picrylhydrzyl (DPPH), absolute ethanol (96%, *v*/*v*) and methanol were purchased from Sigma-Aldrich (St. Louis, MO, USA). Acetonitrile, water, and methanol (HPLC grade) were purchased from Merck (Darmstadt, Germany). Reference HPLC standards, gallic acid, pyrogallol, protocatechuic acid, chlorogenic acid, procyanidin B2, vanilic acid, caffeic acid, syringic acid, epicatechin, *p*-coumric acid, ferulic acid, sinapic acid, rutin, hyperoside, isoquercetin, kaempferol-3-*O*-glukoside, resveratrol, quercetin, kaempferol (purity ≥99%) were purchased from Extrasynthese (Genay, France). 

For metal composition, all chemicals were of analytical grade and were supplied by Merck. All glassware was soaked in 10% HNO_3_ for minimum 12 h and rinsed well with distilled water. Ultra-pure water was prepared by passing doubly de-ionized water from a Milli-Q system. Multi-element stock solution (Alfa Aesar, Thermo Fisher Scientific, Waltham, MA, USA) containing 1.000 g/L of major and trace elements was used to prepare intermediate multi-element standard solutions for ICP-OES measurements. Monosodium phosphate and disodium phosphate (Merck, Darmstadt, Germany), used for preparation of supporting electrolyte for electrochemical measurements, were of analytical grade, and used as received.

#### 3.1.2. Plant Material

The material used in this study were dried herbal parts (bilberry fruits and leaves), collected at Bjelasica Mountain, Montenegro. The voucher specimens (No VMF_121215, and VML_111215) were deposited at the Faculty of Pharmacy, Department of Botany, University of Belgrade, where the identification was performed. Shortly before the extraction, samples were ground in an electric mill (all samples powdered, dimension of the particles 355, as recommended by Ph. Eur. 9).

### 3.2. Extraction and Analytical Methods

#### 3.2.1. Maceration, Infusion and Soxhlet Extraction

The processes of maceration and infusion of milled fruits and leaves were performed according to the processes described below, providing the samples MLEM-*Myrtilli folium* and MFEM-*Myrtilli fructus* extracts and MLEI-*Myrtilli folium* and MFEI-*Myrtilli fructus* extracts, respectively. Applying the classical method of continuous, Soxhlet, extraction provided the samples MLES-*Myrtilli folium* and MFES-*Myrtilli fructus* extracts.

##### Maceration

Ground plant material (50 g, fruits and leaves, each) was mixed with 300 mL of 70% ethanol. The extraction was carried out at room temperature with occasional stirring. After 72 h, sample was filtered through filter paper and precipitate was re-extracted with 20 mL of fresh solvent. After 48 h, the process was repeated. Combined supernatants were evaporated to dryness. The yield of extraction was expressed as a percentage.

##### Soxhlet Extraction

Ten g of the plant material (fruits and leaves) was placed in a Soxhlet thimble in a Soxhlet apparatus and extracted with 325 mL of solvent (70% ethanol) for 72 h at the boiling point of the solvent. After that, the sample was cooled to room temperature and evaporated to dryness. The yield of extraction is expressed as a percentage. 

##### Infusion

Boiling water (150 mL) was poured over 10 g of plant material (fruits and leaves), covered with the watch glass and left to boil further for 2 min. After this time, container was removed from the heater, covered and left to sit for 30 min. Sample was filtered through filter paper, and then evaporated to dryness. The yield of extraction is expressed as a percentage. 

#### 3.2.2. Determination of Total Phenolics, Tannins, Flavonoids, Antocyanins and Procyanidins Content

The total phenolics content was determined by the Folin-Ciocalteu method. One hundred microliters of MeOH solutions of the investigated extracts MLEI, MLEM, MLES, MFEI, MFEM and MFES (0.57, 0.49, 0.56, 0.51, 0.55, 0.62 mg/mL, respectively) was mixed with 0.75 mL of Folin-Ciocalteu reagent (previously diluted 10-fold with distilled water) and allowed to stand at 22 °C for 5 min; 0.75 mL of sodium bicarbonate (60 g/L) solution was added to mixture. After 90 min at 22 °C, absorbance was measured at 725 nm. Gallic acid (0–100 mg/L) was used for calibration of a standard curve. The calibration curve showed the linear regression at r > 0.99, and the results are expressed as milligrams of gallic acid equivalents per gram of plant extracts dry weight (mg GAE/g DW). The content of total phenolics presented the mean of three determinations. 

The percentage content of TT was calculated using the method described in the European Pharmacopoeia 9.0 [39]. Briefly, decoctions prepared from the investigated extracts were treated with phosphomolybdotungstic reagent in alkaline medium after and without treatment with hide powder. The absorbance was measured by a HP 8453 UV-VIS spectrophotometer (Agilent Technologies, Santa Clara, CA, USA), at λ_max_ 760 nm. The percentage of tannins (the mean of three determinations) expressed as pyrogallol (%, *w*/*w*), was calculated from the difference in absorbance of total polyphenols (A_1_) and polyphenols not adsorbed by hide powder (A_2_), using following expression:

62.5(A_1_ − A_2_) × m_2_/(A_3_ − m_1_)

where m_1_ represented mass of the sample to be examined, in grams; and m_2_ mass of pyrogallol, in grams.

The content of TF was calculated using the method described in the European Pharmacopoeia 9.0 [39]. Shortly, the sample was extracted with acetone/HCl under reflux condenser; the AlCl_3_ complex of flavonoid fraction extracted by ethyl acetate was measured by a HP 8453 UV-VIS spectrophotometer, at λ_max_ 425 nm. The content of flavonoid (mean of three determinations), expressed as hyperoside percentage, was calculated using following expression:

A × 1.25/m

where A was absorbance at 425 nm and (m) was mass of the extracts to be examined in grams.

Total anthocyanin content was investigated according to the procedure described in European Pharmacopoeia 9.0 [39]. Shortly, the investigated extract was hydrolysed under reflux with a MeOH/HCl mixture. The absorbance of the solution was measure by a HP 8453 UV-VIS spectrophotometer at 528 nm. The percentage content of anthocyanins (mean of three determinations), expressed as cyanidin-3-glucoside chloride, was calculated using following expression:

A × 5000/(718 × m)

where A was absorbance at 528 nm and m was mass of the extracts to be examined in grams.

The percentage content of procyanidins was calculated using the method described in European Pharmacopoeia 9.0 [39]. Shortly, the investigated extract was hydrolysed under reflux by an EtOH/HCl mixture. Procyanidins were separated with BuOH from the aqueous layer; the absorbance was measured by a HP 8453 UV-VIS spectrophotometer at λ_max_ 545 nm. The percentage content of procyanidins (mean of three determinations), expressed as cyaniding chloride, was calculated using following expression:

A × 500/(75 × m)

where A was absorbance at 545 nm and m was mass of the extracts to be examined in grams.

#### 3.2.3. Determination of Metal Content

Investigated MLEI, MLEM, MLES, MFEI, MFEM and MFES extracts (0.5 g) were transferred into PTFE cuvettes, and 7 mL of 65% HNO_3_ and 1 mL 30% H_2_O_2_ were added. Microwave digestion was performed in a microwave oven (Ethos 1, Advanced Microwave Digestion System, Milestone, Sorisole, Italy). Digestion was performed under following program: warm up for 10 min to 180 °C and held for 15 min at that temperature. After a cooling down period, samples were quantitatively transferred into volumetric flask (50 mL) and diluted with distilled water. Each sample was analyzed in duplicate, and each analysis consisted of three replicates. The measurements of metal content were carried out in a model 6500 Duo Inductively Coupled Atomic Emission Spectrometer (ICP-OES, Thermo Scientific, Waltham, MA, USA).

#### 3.2.4. HPLC Analysis

“Fingerprinting” of the investigated phenolic compounds was achieved by an Agilent Technologies 1200 HPLC system equipped with Lichrospher 100RP 18e column, applying gradient elutions of two mobile phases, i.e., “A/B” (“A”-0.2M solution of phosphoric acid, and “B”-being a pure acetonitrile) at flow–rates 1 mL/min, with photodiode-array (PDA) detection (UV at 260, 325 nm), always within 70 min. Best combinations were 89–75% A (0–35 min); 75–60% A (35–55 min); 60–35% A (55–60 min) and 35–0% A (60–70 min). The concentrations of investigated samples were 0.42, 0.50, 0.50, 0.44, 0.46, 0.44 mg/mL for MLEI, MLEM, MLES, MFEI, MFEM and MFES, respectively. Prior to injection, samples were filtered through PTFE membrane filter. For standard used in the investigation, the concentration were: 0.01 mg/mL for procyanidin B2, 0.15 mg/mL for isoquercetin, 0.26 mg/mL for hyperoside, 0.28 mg/mL for kaempferol-3-*O*-glukoside, 0.30 mg/mL for vanilic and neochlorogenic acid and kaempferol, 0.34 mg/mL for gallic and protocatechuic acid, 0.36 mg/mL for quercetin, 0.38 mg/mL for sinapic acid and resveratrol, 0.40 mg/mL for epicatechin and rutin, 0.46 mg/mL for pyrogallol, 0.52 mg/mL for caffeic acid, 0.56 mg/mL for chlorogenic acid, 0.56 mg/mL for ferulic acid, 0.74 mg/mL for *p*-coumric acid.The volume of standard solutions being injected, as well as for the tested sample extracts, was 4 µL. 

Identification was based on retention times and overlay curves. Once spectra matching succeeded, results were confirmed by spiking with respective standards to achieve a complete identification by means of the so-called peak purity test. Those peaks not fulfilling these requirements were not quantified. Quantification was performed by external calibration with standards.

### 3.3. Methods for Determination of Antioxidant Activity

#### 3.3.1. Radical-Scavenging Activity

Diluted extract (300 µL of the stock solution with concentrations of 0.42, 0.50, 0.50, 0.44, 0.46, 0.44 mg/mL for MLEI, MLEM, MLES, MFEI, MFEM and MFES, respectively) and 2.7 mL of 0.1 mM ethanol DPPH solution were mixed. The absorbance was recorded at 517 nm after 30 min incubation at room temperature in the dark, against ethanol as a blank. Free radical scavenging activity was calculated against to control solution, containing ethanol instead of test solution using the formula:

DPPH radical scavenging capacity (%) = 100 − [(A_S_ − A_B_) × 100/A_C_]

A_S_ was absorption of ethanol solution of the extract treated with DPPH radical solution; A_B_ was absorption of ethanol solution of the extract which was not treated with DPPH radical solution; A_C_ was absorption of ethanol solution of the DPPH. 

The inhibition percentage was plotted against concentration of the samples, and IC_50_ values were determined by linear regression analysis. The synthetic antioxidant *tert*-butyl hydroxytoluene (BHT) were used as positive control.

#### 3.3.2. Ferric-Reducing Antioxidant Power (FRAP)

Diluted extract (100 µL of the stock solution with the concentration 0.42, 0.50, 0.50, 0.44, 0.46, 0.44 mg/mL for MLEI, MLEM, MLES, MFEI, MFEM and MFES, respectively) and 3.0 mL of freshly prepared FRAP reagent (25 mL of 300 mM acetate buffer pH 3.6 plus 2.5 mL of 10 mM TPTZ solution in 40 mM HCl plus 2.5 mL of 20 mM FeCl_3_ × 6H_2_O) were mixed. The absorbance was recorded at 593 nm against a blank, containing 100 µL of resembling solvent, after 30 min incubation at 37 °C. The FRAP-value was calculated from the calibration curve of FeSO_4_ × 7H_2_O standard solutions, covering the concentration range 100–1000 mmol/L and expressed as mmol Fe^2+^/g extracts.

#### 3.3.3. Cyclic Voltammetry

The cell (10 mL) consisted of a three-electrode system, a boron-doped diamond (BDD) electrode (inner diameter of 3 mm; Windsor Scientific Ltd., Slough, UK) embedded in a polyether ether ketone (PEEK) body with an inner diameter of 3 mm, a resistivity of 0.075 Ω cm and a boron doping level of 1000 ppm (as declared by the supplier) was used as the working electrode, an Ag/AgCl (3 M KCl) as a reference electrode and a Pt wire as a counter electrode. Prior to start the first measurement, the BDD electrode was rinsed with deionised water and gently rubbed with a piece of damp silk cloth until a mirror-like appearance of surface was obtained (with minimal probability of mechanical damage of surface). Subsequently, it was anodically pre-treated by setting +2 V during 180 s in 1 M H_2_SO_4_ in order to clean the electrode surface (get rid of any impurities) followed by cathodic pre-treatment at −2 V during 180 s to renew the hydrogen terminated surface of the working electrode. In order to confirm stability and advantages of the BDD electrode before starting measurements and at the end of working day, potential/current changes in the K_4_[Fe(CN)_6_]/K_3_[Fe(CN)_6_] couple was monitored. It was observed that during day these changes are lower than 5%. Samples were studied in phosphate buffer at pH 7 because the oxidation of phenolics at neutral pH mimics physiological conditions. The scan was taken in the potential range between 0 mV and 1200 mV with a scan rate 100 mV s^−1^.

### 3.4. Statistical Analysis

All assays were performed in triplicate for each extracts. Statistical analyses were performed using the Microsoft Excel 2007 (Microsoft Corporation, Redmond, WA, USA) and SPSS Statistics 22.0 (IBM Corporation, Armonk, NY, USA) program packages. The data were analyzed using the analysis of variance (ANOVA) and Tukey’s HSD test as a post hoc test. Correlation coefficient was calculated by regression and correlation analysis. 

## 4. Conclusions

Our results revealed that all investigated bilberry leaves and fruits extracts were rich in phenolic compounds and demonstrated good antioxidant activity measured by using two spectrophotometric methods, whose results were in accordance with the cyclic voltammetry tests performed as well. This research provided systematic and thorough information on the phenolic compounds in the extracts obtained by extraction methods using different solvents. The results pointed out that ethanol might be considered a supreme solvent in regard to water, when antioxidant properties and total phenolic content were taken into account. Using a HPLC method, we confirmed the presence of eighteen individual phenolic compounds in different amounts in the leaves and fruits extracts. The most abundant was chlorogenic acid, followed by protocatechuic acid, with resveratrol, isoquercetin, quecetin and hyperoside present in significant quantities in investigated extracts. The identified phenolic compounds might take part in reducing the risk of type 2 diabetes and the associated increased risk of microvascular and macrovascular complications, according to the available literature data. However, investigated water extracts contained the higher quantity of the majority of essential metals in comparison to the ethanol extracts. Such a presence of the metals in the investigated samples might be recognized as beneficial for insulin level regulation; the results were especially promising for Mn. These findings contribute to explain the traditionally established use of bilberry as a plant with antidiabetic effects. Based on the presented data, the extraction methods applied revealed that the obtained extracts contained significant amount of the valuable substances recognized for their antiglycemic potential. Further investigation might focus on providing data regarding the pharmacological activity of the extracts obtained by the method which might provide optimized quantity of both, the phenolics and mineral components. Our results indicated that, in addition to their traditional usage in folk medicine, this plant might represent a valuable source of natural antioxidants, and thus may be considered as a great potential for the food industry, representing feasible alternatives to synthetic additives.

## Supplementary Materials

Supplementary Materials are available online.

## Abbreviations

2,4,6-tripyridyl-s-triazine (TPTZ), hydrochloric acid (HCl), iron (III) chloride (FeCl_3_), 2,6-di-*tert*-butyl-4-methylphenol (BHT), *n*-butanol (BuOH), 1,1′-diphenyl-2-picrylhydrzyl (DPPH), volume solute per total volume of solution (*v*/*v*), high-performance liquid chromatography (HPLC), percentage (%), gram per litre (g/L), Inductively Coupled Atomic Emission Spectrometer (ICP-OES), *Myrtilli folium* extract obtained by Soxhlet (MLES), *Myrtilli fructus* extract obtained by Soxhlet (MFES), *Myrtilli folium* extract obtained by maceration (MLEM), *Myrtilli fructus* extract obtained by maceration (MFEM), *Myrtilli folium* extract obtained by infusion (MLEI), *Myrtilli fructus* extract obtained by infusion (MFEI), European Pharmacopoeia 9.0 (Ph. Eur. 9), milliliter (mL), gram (g), hour (h), minute (min), methanol (MeOH), milligram per milliliter (mg/mL), Celsius scale (°C), nanometer (nm), milligram per litre (mg/L), gallic acid equivalents per gram of plant extracts dry weight (mg GAE/g DW), coefficient of determination or the coefficient of multiple determination for multiple regression (R^2^), wavelength (λ), maximum (max), weight solute per total weight of solution (*w*/*w*), absorbance (A), mass (m), aluminium chloride (AlCl_3_), total phenolics (TP), total tannins (TT), total flavonoid (TF) content, ethanol (EtOH), polytetrafluoroethylene (PTFE), nitric acid (HNO_3_), hydrogen peroxide (H_2_O_2_), milliliter per minute (mL/min), photodiode-array (PDA), microliter (µL), *tert*-butyl hydroxytoluene (BHT), concentration at which 50% of radicals are scavenged (IC_50_), ferric reducing antioxidant power (FRAP), iron(II) sulfate (FeSO_4_), millimoles per liter (mmol/L), boron-doped diamond (BDD), polyether ether ketone (PEEK), milimetre (mm), ohm (Ω), parts per million (ppm), statistical significance (p), silver (Ag), silver-chloride (AgCl), potassium-chloride (KCl), platinum (Pt), volt (V), second (s), sulfuric acid (H_2_SO_4_), molarity (M), potassium hexacyanoferrate(II) trihydrate (K_4_[Fe(CN)_6_]), potassium hexacyanoferrate(III) trihydrate (K_3_[Fe(CN)_6_]), millivolts per second (mv/s), cyclic voltammetry (CV), milligram per gram (mg/g), glucose transporter (GLUT), World Health Organization (WHO), daily mineral intakes (DMI), sodium (Na), 5′-adenosine monophosphate-activated protein (AMPK), microgram per gram (µg/g), reactive oxygen species (ROS), reactive nitrogen species (RNS), superoxide (O^2−^), hydroxyl (OH), nitric oxide (NO), nitrogen dioxide (NO_2_^−^), peroxynitrite (OONO^−^), reactive species (RS), superoxide dismutase (SOD), glutathione peroxidase (GPx), catalase (CAT), microgram per milliliter (µg/mL), millimoles per gram (mmol/g), principal component analysis (PCA), current (I), ampere (A), potential (E), aluminum (Al), barium (Ba), calcium (Ca), potassium (K), magnesium (Mg), strontium (Sr), iron (Fe), nickel (Ni), chromium (Cr), copper (Cu), cobalt (Co), arsenic (As), lead (Pb), manganese (Mn), cadmium (Cd), zinc (Zn).
