# Supplementary material for: Wild Bilberry (Vaccinium myrtillus L., Ericaceae) from Montenegro as a Source of Antioxidants for Use in the Production of Nutraceuticals
Source: Molecules. 2018 Jul 26;23(8):1864. doi: 10.3390/molecules23081864 (PMC6222815; doi:10.3390/molecules23081864)
Supplement: Supplementary file 1 [file molecules-23-01864-s001.pdf]

molecules

Supplementary Materials

Wild bilberry (*Vaccinium myrtillus* L., Ericaceae) from Montenegro as a Source of Antioxidants for Use in the Production of Nutraceuticals

Snezana Brasanac-Vukanovic[1], Jelena Mutic[2], Dalibor M. Stankovic[2,3], Ivana Arsic[4], Nada Blagojevic[1], Vesna Vukasinovic-Pesic[1] and Vanja M. Tadic[5,*]

[1] Faculty of Metallurgy and Technology, University of Montenegro, Dzordza Vasingtona bb, 20000 Podgorica, Montenegro; e-mails: sneza_b@yahoo.com; nadab@ac.me; vesnav@ac.me
[2] Faculty of Chemistry, University of Belgrade, Studentski trg 12-16, 11001, Belgrade, Serbia; e-mail: jmutic@chem.bg.ac.rs
[3] The Vinča Institute of Nuclear Sciences, University of Belgrade, POB 522, 11001 Belgrade, Serbia; e-mail: dalibors@chem.bg.ac.rs
[4] University of Nis, Faculty of Medicine, Department of Pharmacy, 18000 Nis, Serbia; e-mail: ivanafarmacija@gmail.com
[5] Institute for Medicinal Plant Research "Dr Josif Pancic", Tadeusa Koscuska 1, 11000, Belgrade, Serbia; e-mail: vtadic@mocbilja.rs
* e-mail: vtadic@mocbilja.rs ; Tel.: +381 11 3031 658; +381 64 3130 918; Fax: +381 11 3031 655

Contents

Table S1. Content of phenolic compounds determined by HPLC in the investigated extracts

Figure S1. Identified compounds in the investigated extracts: **(a)** derivatives of hydroxybenzoic acid; **(b)** derivatives of hydroxycinnamic acid; **(c)** flavons and flavonols; **(d)** flavanols; **(e)** pyrogallol; **(f)** resveratrol (stilbene)

Table S2a. The metal content in the investigated extracts

Table S2b. The daily mineral intake of essential and toxic elements [%] calculated in 1g of the investigated extracts according to recommendation*

Figure S2. Principal component loading plot of **(a)** leaves; and **(b)** fruits extracts. Total phenolic compounds (TP), total tannins (TT), total flavonoids (TF), total procyanidins, total anthocyanins, essential metals content, toxic metals content, radical scavenging activity-IC_{50} (DPPH), ferric reducing antioxidant power (FRAP)

Table S3. Correlation between TT, TF, proanthocyanidins, anthocyanins and TP and antioxidant activity; correlation between the applied tests DPPH and FRAP in the leaves and fruits extracts

Table S4. Correlation between identified individual phenolic compounds and antioxidant activity in the leaves and fruits extracts

Table S5. Correlation between all the identified metals and antioxidant activity in the leaves and fruits extracts

Table S6. Correlation between the group of essential and toxic metals and antioxidant activity in the leaves and fruits extracts

Table S1. Content of phenolic compounds determined by HPLC in the investigated extracts [mg/g].

Phenolic compounds		MFEI	MFEM	MFES	MLEI	MLEM	MLES
**Gallic acid**	1	0.73	1.03	0.78	0.58	0.80	0.54
**Pyrogallol**	2	-	-	-	3.16	2.45	3.46
**Neochlorogenic acid**	3	0.01	0.41	-	1.86	0.34	3.72
**Protocatechuic acid**	4	1.10	1.42	1.23	1.74	1.40	1.73
**Chlorogenic acid**	5	1.82	2.48	1.95	59.70	45.51	55.28
**Procyanidin B2**	6	0.11	0.13	0.14	0.89	1.03	0.31
**Caffeic acid**	7	0.16	0.31	0.32	1.25	1.95	1.90
**Epicatechin**	8	-	-	-	4.38	5.16	5.75
**p-coumric acid**	9	0.21	0.34	0.24	1.53	1.26	2.08
**Sinapic acid**	10	0.03	0.06	0.03	0.39	0.18	0.63
**Ferulic acid**	11	0.05	0.07	0.01	0.11	0.26	0.28
**Stilbenoid derivative 1**	12	*	*	*	*	*	*
**Rutin**	13	-	-	-		4.73	4.94
**Hyperoside**	14	0.17	0.34	0.26	2.55	2.51	2.38
**Isoquercetin**	15	0.30	0.30	0.31	16.20	14.62	9.92
**Stilbenoid derivative 2**	16	*	*	*	*	*	*
**Stilbenoid derivative 3**	17	*	-	*	*	*	*
**Kaempferol-3-*O*-glukoside**	18	0.05	0.15	0.06	1.60	1.56	1.38
**Quercetin derivative 1**	19	*	-	*	*	*	*
**Quercetin derivative 2**	20	*	-	*	-	-	-
**Resveratrol**	21	0.01	0.07	0.03	4.69	4.60	5.15
**Chlorogenic acid derivative**	22	-	-	-	-	*	-
**Quercetin**	23	0.07	0.40	0.46	1.16	2.11	7.27
**Kaempferol**	24	-	-	-	0.03	0.10	0.26

* tentative identification

(a)

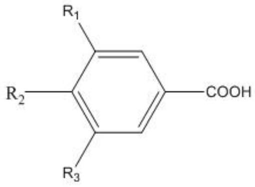

**Derivatives of hydroxybenzoic acid**	**R_1**	**R_2**	**R_3**
Gallic acid	OH	OH	OH
Protocatechuic acid	H	OH	OH

(b)

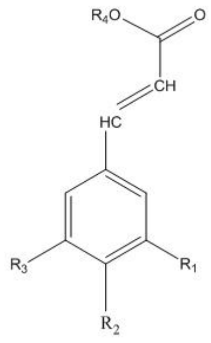

**Derivatives of hydroxycinnamic acid**	**R1**	**R2**	**R3**	**R4**
Neochlorogenic acid (5-caffeoylquinic acid)	OH	OH	H	Quinic acid 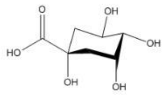
Chlorogenic acid (3-caffeoylquinic acid)	OH	OH	H	Quinic acid 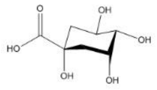
Caffeic acid	OH	OH	H	H
p-Coumaric acid	H	OH	H	H
Sinapic acid	OCH_3	OH	OCH_3	H
Ferulic acid	OCH_3	OH	H	H

(c)

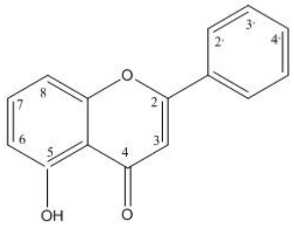

**Flavones and flavonols**	C_3	$C_{3'}$	$C_{4'}$	C_7	C_8
Rutin	*O*-*β*-D-rutinosyl	OH	OH	OH	H
Hyperoside	*O*-*β*-D-galactosyl	OH	OH	OH	H
Isoquercetin	*O*-*β*-D-glucosyl	OH	OH	OH	H
Kaempferol-3-*O*-glukoside	*O*-*β*-D-glucosyl	H	OH	OH	H
Quercetin	OH	OH	OH	OH	H
Kaempferol	OH	H	OH	OH	H

(d)

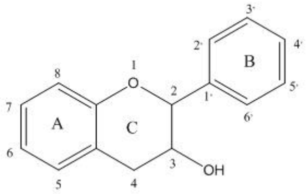

**Flavanols**	**C_5**	**C_4**	**C_7**	**$C_{4'}$**	**$C_{5'}$**
procyanidin B2	OH	epicatechin 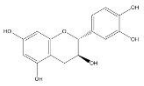	OH	OH	OH
epicatechin	OH	H	OH	OH	OH

(e) Pyrogallol

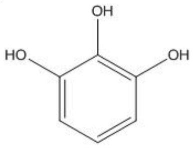

(f) Resveratrol (stilbene)

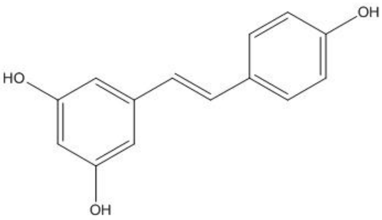

Figure S1. Identified compounds in the investigated extracts: **(a)** derivatives of hydroxybenzoic acid; **(b)** derivatives of hydroxycinnamic acid; **(c)** flavons and flavonols; **(d)** flavanols; **(e)** pyrogallol; **(f)** resveratrol (stilbene).

Table S2a. The metal content in the investigated extracts.

	MFES	MFEM	MFEI	MLES	MLEM	MLEI
**Al (µg/g)**	14.2±0.7	8.92±0.04	40.30±0.06	7.45±0.06	17.8±0.2	186.2±0.2
**As (µg/g)**	-	-	0.039±0.002	0.059±0.002	0.297±0.004	-
**Ba (µg/g)**	2.626±0.008	1.101±0.006	11.43±0.02	5.36±0.03	6.283±0.009	69.6±0.8
**Cd (µg/g)**	0.0249±0.0002	0.026±0.002	0.030±0.002	0.047±0.002	0.016±0.001	0.112±0.007
**Co (µg/g)**	-	0.072±0.002	-	-	0.055±0.001	-
**Cr (µg/g)**	0.181±0.004	0.22±0.03	0.305±0.008	0.463±0.009	0.123±0.002	1.11±0.03
**Cu (µg/g)**	3.62±0.03	3.32±0.05	1.513±0.010	33.31±0.09	19.53±0.04	2.99±0.04
**Mn (µg/g)**	42.85±0.10	43.67±0.06	150.3±0.7	472±4	1210±2	251.4±11
**Ni (µg/g)**	0.96±0.02	0.94±0.02	1.469±0.008	2.184±0.005	4.398±0.006	2.85±0.02
**Pb (µg/g)**	0.104±0.002	0.486±0.007	0.096±0.004	0.63±0.02	0.60±0.05	0.47±0.02
**Sr (µg/g)**	0.582±0.003	0.738±0.007	3.675±0.008	5.96±0.02	11.775±0.009	14.67±0.08
**Zn (µg/g)**	8.99±0.02	6.281±0.007	17.35±0.05	29.55±0.09	20.49±0.04	31.48±0.04
**Na (µg/g)**	107±1	33.6±0.3	951±15	1262±7	1128±20	138±2
**Fe (µg/g)**	11.9±0.2	5.90±0.05	37.6±0.2	17.37±0.04	25.75±0.09	19.8±0.2
**K (mg/g)**	6.60±0.08	6.01±0.10	10.66±0.10	8.19±0.02	9.24±0.02	17.22±0.02
**Mg (mg/g)**	0.442±0.009	0.397±0.007	1.024±0.003	1.546±0.009	2.66±0.02	3.83±0.02
**Ca (mg/g)**	0.425±0.001	0.383±0.005	1.321±0.010	1.233±0.004	2.97±0.03	5.94±0.02

*Detection limit of the instrument for Co and As was 0.002 µg/g; 0.005 µg/g, respectively

Table S2b. The daily mineral intake of essential and toxic elements [%] calculated in 1g of the investigated extracts according to recommendation*.

	MDI** (µg per day) and RDA*** (mg per day)	MFES	MFEM	MFEI	MLES	M LEM	MLEI
**Cr**	0.04	0.45	0.55	0.76	1.16	0.31	2.78
**K**	2000	0.33	0.30	0.53	0.41	0.46	0.86
**Mg**	375	0.11	0.12	0.27	0.41	0.71	1.02
**Ca**	800	0.05	0.05	0.17	0.15	0.37	0.74
**Cu**	1	0.36	0.33	0.15	3.33	1.95	0.30
**Mn**	2	2.14	2.18	7.52	23.60	60.50	12.57
**Zn**	10	0.09	0.06	0.17	0.30	0.20	0.31
**Fe**	14	0.09	0.04	0.27	0.12	1.84	0.14
**Pb*** **	250	0.04	0.19	0.04	0.25	0.24	0.19
**As*** **	150	-	-	0.03	0.04	0.20	-
**Cd*** **	25	0.10	0.10	0.12	0.19	0.06	0.45

* The used literature [1-4];

[1-4] **MDI-Maximum daily intake for essential elements; ***RDA- recommended daily allowance for toxic elements

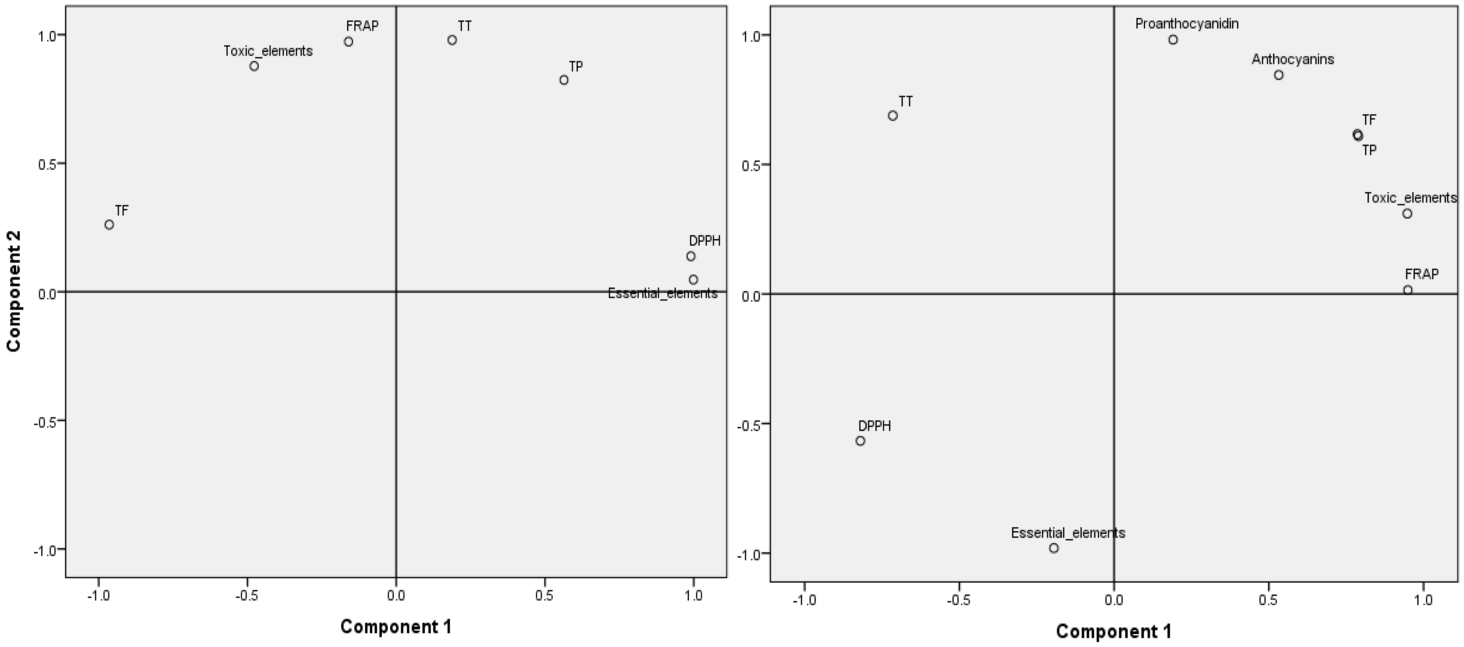

(a) **(b)**

Figure S2. Principal component loading plot of **(a)** leaves; and **(b)** fruits extracts. Total phenolic compounds (TP), total tannins (TT), total flavonoids (TF), total procyanidins, total anthocyanins, essential metals content, toxic metals content, radical scavenging activity-IC_{50} (DPPH), ferric reducing antioxidant power (FRAP).

Table S3. Correlation between TT, TF, proanthocyanidins, anthocyanins and TP and antioxidant activity; correlation between the applied tests DPPH and FRAP in the leaves and fruits extracts.

	**Fruits**		**Leaves**	
	**DPPH (R^2)**	**FRAP (R^2)**	**DPPH (R^2)**	**FRAP (R^2)**
**TT**	0.0434	0.4898	0.104	0.882
**TF**	0.996	0.6517	0.8446	0.1719
**TP**	0.9972	0.6617	0.4529	0.5249
**Proanthocyanidins**	0.5059	0.0491		
**Anthocyanins**	0.8381	0.3085		
**DPPH**		0.710		0.0006

Table S4. Correlation between identified individual phenolic compounds and antioxidant activity in the leaves and fruits extracts.

	Fruits		Leaves	
**Phenolic compounds**	**DPPH**	**FRAP**	**DPPH**	**FRAP**
**Gallic acid**	y=-0.0005x+0.1294 R^2=0.9947	y=24.839x+0.0641 R^2=0.7743	y=0.0008x+0.0567 R^2=0.0696	y=13.131x+0.0389 R^2=0.9178
**Pyrogallol**	-	-	y=-0.0047x+0.3386 R^2=0.1721	y=-45.575x+0.3856 R^2=0.8095
**Neochlorogenic acid**	y=-0.0008x+0.0808 R^2=0.9528	y=39.847x-0.0178 R^2=0.8833	y=-0.2414x+3.8452 R^2=0.4201	y=-109.41x+40.3745 R^2=0.6885
**Protocatechuic acid**	y=-0.0006x+0.1713 R^2=0.9652	y=21.234x+0.1081 R^2=0.5299	y=-0.0042x+1.6555 R^2=0.0095	y=-188.02x+1.968 R^2=0.9853
**Chlorogenic acid**	y=-0.0012x+0.3084 R^2=1	y=52.581x+0.1656 R^2=0.7087	y=0.0294x+5.1214 R^2=0.0339	y=-701.23x+6.6349 R^2=0.9742
**Procyanidin B2**	y=-0.0002x+0.1435 R^2=0.1449	y=-4.8387x+0.1305 R^2=0.0311	y=0.0701x+0.2002 R^2=0.6956	y=198.72x+0.379 R^2=0.2827
**Caffeic acid**	y=-0.0002x+0.0404 R^2=0.3273	y=0.6452x+0.0255 R^2=0.0017	y=-0.0066x+0.2209 R^2=0.5748	y=25.99x+0.122 R^2=0.4488
**Epicatechin**	-	-	y=-0.0149x+0.6249 R^2=0.9578	y=15.511x+0.4809 R^2=0.0524
***p*-Coumaric acid**	y=-0.0002x+0.0441 R^2=0.9982	y=9.1935x+0.0186 R^2=0.6718	y=-0.0068x+0.2147 R^2=0.5654	y=-25.958x+0.2093 R^2=0.4111
**Ferulic acid**	y=-5x10^{-5} x+0.0078 R^2=0.2412	y=4.1935x+0.0006 R^2=0.7788	y=-0.0018x+0.0343 R^2=0.7363	y=4.8562x+0.0118 R^2=0.285
**Sinapic acid**	y=-5x10^{-5} x+0.0077 R^2=0.9824	y=2.0968x+0.0017 R^2=0.5841	y=-0.0033x+0.0646 R^2=0.4124	y=-16.933x+0.0704 R^2=0.5641
**Rutin**	-	-	y=0.0035x+0.4714 R^2=0.2866	y=-25.096x+0.5447 R^2=0.7347
**Hyperoside**	y=-0.0003x+0.0487 R^2=0.8778	y=9.1935x+0.0183 R^2=0.3622	y=0.0018x+0.2335 R^2=0.9512	y=1.6454x+0.2447 R^2=0.039
**Isoquercetin**	y=8x10^{-6} x+0.0295 R^2=0.4391	y=-0.6016x+0.0307 R^2=0.9243	y=0.071x+0.8067 R^2=0.9754	y=42.636x+1.2792 R^2=0.0178
**Kaempferol-3-*O* -glucoside**	y=-0.0002x+0.0255 R^2=0.9861	y=9.8387x+0.0001 R^2=0.811	y=0.0025x+0.1319 R^2=0.9487	y=2.3323x+0.1471 R^2=0.0413
**Resveratrol**	y=-0.0001x+0.0126 R^2=0.998	y=5x-0.0007 R^2=0.75	y=-0.0056x+0.5244 R^2=0.7266	y=-14.792x+0.5078 R^2=0.2524
**Quercetin**	y=-0.0004x+0.0634 R^2=0.3009	y=0.4839x+0.0296 R^2=0.0002	y=-0.07x+0.8939 R^2=0.9355	y=-74.329x+0.4873 R^2=0.0533
**Kaempferol**	-	-	y=-0.0026x+0.032 R^2=0.9903	y=-0.8626x+0.0136 R^2=0.0056

Table S5. Correlation between all the identified metals and antioxidant activity in the leaves and fruits extracts.

	**Leaves**	**Leaves**	**Fruits**	**Fruits**
	**IC_{50}(μg/mL)**	**Fe^{2+} (mmol/g)**	**IC_{50}(μg/mL)**	**Fe^{2+}(mmol/g)**
**Al (μg/g)**	y=18.279x-71.24 R^2=0.6853	y=-57033x+175.04 R^2=0.337	y=0.4501x-16.41 R^2=0.5958	y=-9319.4x+28.595 R^2=0.0954
**As (μg/g)**	y=0.0023x+0.1361 R^2=0.0042	y=153.8x-0.1633 R^2=0.9983	y=0.0005x-0.0302 R^2=0.4387	y=-6.2903x+0.018 R^2=0.0242
**Ba (μg/g)**	y=6.5254x-23.513 R^2=0.6485	y=22060x+67.525 R^2=0.3744	y=0.1468x-7.1945 R^2=0.5755	y=-2895.8x+7.369 R^2=0.0836
**Cd (μg/g)**	y=0.0061x+0.011 R^2=0.3205	y=-40.176x+0.132 R^2=0.7016	y=5x10^{-5}x+0.0231 R^2=0.2448	y=0.2419x+0.0268 R^2=0.0025
**Co (μg/g)**	y=0.0009x+0.0117 R^2=0.0152	y=30.751x-0.038 R^2=0.9784	y=-0.0014x+0.142 R^2=0.9604	y=69.677x-0.0317 R^2=0.871
**Cr (μg/g)**	y=0.0603x+0.0982 R^2=0.2983	y=-417.48x+1.3307 R^2=0.7233	y=0.0009x+0.1619 R^2=0.1599	y=-3.5004x+0.2412 R^2=0.0126
**Cu (μg/g)**	y=-3.2888x+44.109 R^2=0.9693	y=2951.1x+13.2 R^2=0.0394	y=-0.0221x+4.6593 R^2=0.3114	y=162.1x+2.5475 R^2=0.0834
**Mn (μg/g)**	y=218.52x-295.57 R^2=0.9224	y=-305016x+1957.9 R^2=0.0908	y=1.4101x+38.695 R^2=0.4322	y=-16537x+92.17 R^2=0.0222
**Ni (μg/g)**	y=0.1022x+2.3519 R^2=0.1671	y=1003.8x+1.3037 R^2=0.8148	y=0.0071x+0.5266 R^2=0.472	y=-101.45x+1.2042 R^2=0.0355
**Pb (μg/g)**	y=-0.0167x+0.696 R^2=0.795	y=39.457x+0.4943 R^2=0.2246	y=-0.0076x+0.8633 R^2=0.9671	y=370.97x-0.0681 R^2=0.8587
**Sr (μg/g)**	y=0.9738x+3.2513 R^2=0.9954	y=190.18x+10.453 R^2=0.0019	y=0.038x-1.5043 R^2=0.3946	y=-347.9x+1.9433 R^2=0.0124
**Zn (μg/g)**	y=0.0538x+26.756 R^2=0.0017	y=-5743.8x+37.704 R^2=0.9997	y=0.1642x-2.8232 R^2=0.672	y=-1746.7x+13.785 R^2=0.3778
**Na (μg/g)**	y=-116.02x+1742.2 R^2=0.7376	y=320096x+255.82 R^2=0.2836	y=12.642x-690.74 R^2=0.5106	y=-207069x+529.55 R^2=0.0512
**Fe (μg/g)**	y=0.3775x+18.047 R^2=0.1583	y=3831.3x+13.949 R^2=0.8239	y=0.4589x-19.82 R^2=0.6166	y=-4710.2x+26.317 R^2=0.3226
**K (mg/g)**	y=0.9317x+4.3265 R^2=0.7351	y=-2586.4x+16.292 R^2=0.2862	y=0.0655x+2.2945 R^2=0.5553	y=-1225.8x+8.7373 R^2=0.0727
**Mg (mg/g)**	y=0.2489x+0.7486 R^2=0.9812	y=-179.84x+3.0084 R^2=0.0259	y=0.0083x-0.0883 R^2=0.4387	y=-101.13x+0.6869 R^2=0.0242
**Ca (mg/g)**	y=0.8083x-1.8862 R^2=0.8258	y=-1737.4x+7.5662 R^2=0.1927	y=0.0127x-0.3513 R^2=0.4782	y=-185.16x+0.8578 R^2=0.0379

Table S6. Correlation between the group of essential and toxic metals and antioxidant activity in the leaves and fruits extracts.

	Fruits		Leaves	
	DPPH (R^2)	FRAP (R^2)	DPPH (R^2)	FRAP (R^2)
essential metals	0.5219	0.0563	0.8563	0.1625
toxic metals	0.921	0.9221	0.1234	0.8933

References

1. European Economic Community (EEC). Commission Directive 2008/100/EC. Amending Council Directive 90/496/EEC on nutrition labelling for food stuffs as regards recommended daily allowances, energy conversion factors and definitions. *Off. J. Eur. Commun*. **2008**, *L285*, 9–12. Available online: https://eur-lex.europa.eu/legal-content/EN/TXT/?uri=CELEX:32008L0100 (accessed on 25/may/2018).
2. European Food Safety Authority (EFSA). Dietary exposure to inorganic arsenic in the European population. *EFSA J.* **2014**, *12*, 3597, doi:10.2903/j.efsa.2014.3597.
3. EFSA. Cadmium dietary exposure in the European population. *EFSA J.* **2012**, *10*, 2551, doi:10.2903/j.efsa.2012.2551.
4. EFSA. Scientific opinion on lead in food. EFSA Panel on Contaminants in the Food Chain (CONTAM). *EFSA J.* **2010**, *8*, 1570, doi:10.2903/j.efsa.2010.1570.
